# The TOPSY pessary self-management intervention for pelvic organ prolapse: a study protocol for the process evaluation

**DOI:** 10.1186/s13063-020-04729-w

**Published:** 2020-10-08

**Authors:** Carol Bugge, Rohna Kearney, Melanie Dembinsky, Aethele Khunda, Margaret Graham, Wael Agur, Suzanne Breeman, Lucy Dwyer, Andrew Elders, Mark Forrest, Kirsteen Goodman, Karen Guerrero, Christine Hemming, Helen Mason, Doreen McClurg, Lynn Melone, John Norrie, Ranee Thakar, Suzanne Hagen

**Affiliations:** 1 Faculty of Health Sciences and Sport, University of Stirling, Stirling, UK; 2 The Warrell Unit, St. Mary’s Hospital, Manchester University Hospitals NHS Foundation Trust, Manchester Academic Health Science Centre, Manchester, UK; 3 Faculty of Biology Medicine and Health, School of Medical Sciences, University of Manchester, Manchester, UK; 4 South Tees Hospitals NHS Foundation Trust, James Cook University Hospital, Middlesbrough, UK; 5 NHS Ayrshire & Arran, Crosshouse Hospital, School of Medicine, Dentistry & Nursing, University of Glasgow, Kilmarnock, UK; 6 Health Services Research Unit (HSRU), University of Aberdeen, Aberdeen, UK; 7 Nursing, Midwifery and Allied Health Professions Research Unit, Glasgow Caledonian University, Glasgow, UK; 8 Department of Urogynaecology, NHS Greater Glasgow & Clyde, Glasgow, UK; 9 Grampian University Hospitals NHS Trust, Aberdeen Maternity Hospital & Aberdeen Royal Infirmary, Aberdeen, UK; 10 Yunus Centre for Social Business and Health, Glasgow Caledonian University, Glasgow, UK; 11 Usher Institute of Population Health Sciences and Informatics, College of Medicine and Veterinary Medicine, The University of Edinburgh, Edinburgh, UK; 12 Croydon Health Services NHS Trust, Croydon University Hospital, Croydon, UK

**Keywords:** Process evaluation, Prolapse, Pessary, Self-management, Randomised controlled trial

## Abstract

**Background:**

Process evaluations have become a valued component, alongside clinical trials, of the wider evaluation of complex health interventions. They support understanding of implementation, and fidelity, related to the intervention and provide valuable insights into what is effective in a practical setting by examining the context in which interventions are implemented. The TOPSY study consists of a large multi-centre randomised controlled trial comparing the effectiveness of pessary self-management with clinic-based care in improving women’s condition-specific quality of life, and a nested process evaluation. The process evaluation aims to examine and maximise recruitment to the trial, describe intervention fidelity and explore participants’ and healthcare professionals’ experiences.

**Methods:**

The trial will recruit 330 women from approximately 17 UK centres. The process evaluation uses a mixed-methods approach. Semi-structured interviews will be conducted with randomised women (18 per randomised group/*n* = 36), women who declined trial participation but agreed to interview (non-randomised women) (*n* = 20) and healthcare professionals recruiting to the trial (*n* ~ 17) and delivering self-management and clinic-based care (*n* ~ 17). The six internal pilot centres will be asked to record two to three recruitment discussions each (total *n* = 12–18). All participating centres will be asked to record one or two self-management teaching appointments (*n* = 30) and self-management 2-week follow-up telephone calls (*n* = 30). Process data (quantitative and qualitative) will be gathered in participant completed trial questionnaires. Interviews will be analysed thematically and recordings using an analytic grid to identify fidelity to the intervention. Quantitative analysis will be predefined within the process evaluation analysis plan.

**Discussion:**

The wide variety of pessary care delivered across the UK for women with pelvic organ prolapse presents specific localised contexts in which the TOPSY interventions will be implemented. Understanding this contextual variance is central to understanding how and in what circumstances pessary self-management can be implemented (should it be effective). The inclusion of non-randomised women provides an innovative way of collecting indispensable information about eligible women who decline trial participation, allowing broader contextualisation and considerations of generalisability of trial findings. Methodological insights from examination of recruitment processes and mechanisms have the potential to inform recruitment mechanisms and future recruitment strategies and study designs.

**Trial registration:**

ISRCTN62510577. Registered on 6 October 2017.

## Background

Contemporary trial design advocates the inclusion of a process evaluation alongside a randomised controlled trial (RCT) to support evaluation of complex interventions [1]. The inclusion of a process evaluation, especially in multi-centre, pragmatic trials, has the potential to identify variation in intervention delivery across and within different centres (fidelity), examine the mechanisms of action of the intervention; outline the contexts in which an intervention is implemented and aid understanding of the perspectives of those who deliver and receive the intervention [1–6]. The TOPSY study includes a RCT (with an internal pilot), an economic evaluation of cost-effectiveness and a nested mixed-method process evaluation.

The trial evaluates the clinical and cost-effectiveness of self-management of vaginal pessaries to treat pelvic organ prolapse, compared to clinic-based care (clinic-based follow-up) to improve women’s quality of life. Pelvic organ prolapse affects approximately 40% of women over the age of 40 years [7] and is associated with a range of distressing symptoms which negatively impact upon a woman’s quality of life [8]. Two thirds of women will opt to try a vaginal pessary for prolapse treatment when it is offered [9]. Currently, the most common service model for women treated with a vaginal pessary in the UK is to routinely return to clinic to have their pessary changed, i.e. clinic-based care [10]. No trials have been identified that evaluate the effectiveness of self-management in comparison to clinic-based care in improving women’s quality of life when they use a pessary for the treatment of prolapse.

The self-management intervention evaluated within TOPSY is described in detail in the trial protocol paper [companion paper, manuscript reference TRLS-D-19-01151R1 [11]]. In summary, women are taught how to manage their own pessary by a trained healthcare professional, who has experience in pessary care, in a 30-min teaching appointment. There is one follow-up phone call 2 weeks after the teaching appointment to support self-management and women also have a contact number to call if they should experience difficulties at any time. Women also receive an information leaflet about pessary self-management. The hypothesised mechanism of action that connects the self-management intervention and the outcome of increased quality of life is that self-management increases women’s self-efficacy [10]. Women in the clinic-based care group receive treatment as usual and will be seen at regular intervals as per local centre protocol (usually every 6 months) to have their pessary removed and a new one inserted by a trained healthcare professional.

The process evaluation was developed based on guidance within the Medical Research Council (MRC) Framework for Process Evaluation [1] and recommendations made by the QuinteT group in relation to recruitment [6, 12–14]. The potential for the self-management intervention to be more effective than clinic-based care is reliant on successful intervention delivery, and the individual and structural contexts that surround intervention delivery and implementation. The process evaluation gathers data from women in the self-management and clinic-based care groups in order to understand how these features are operationalised in the real world of clinical practice and individuals’ lives. For example, understanding contextual factors for women who are implementing self-management at home or contextual factors that influence the processes through which women get to clinics to receive care.

In this paper, the protocol elements relating to the process evaluation of the TOPSY study are presented. A companion paper describes the protocol for the trial including the economic evaluation [manuscript reference TRLS-D-19-01151R1 [11]].

## Methods

### Research question and objectives for the process evaluation

#### Research question

What are the barriers and facilitators to intervention acceptability, intervention effectiveness, fidelity to delivery, and adherence for women treated with vaginal pessary and the health professionals who treat them, and how does this differ between randomised groups?

#### Objectives

The objectives of undertaking a nested process evaluation are to:

1. Maximise recruitment (data gathered during the internal pilot only);
2. Investigate reasons why eligible women declined trial participation;
3. Understand women’s and healthcare professionals’ experiences of the interventions and views on acceptability;
4. Assess adherence to allocated trial group;
5. Describe fidelity to intervention delivery; and
6. Identify contextual factors that may interact with intervention effectiveness.

### Overview of methods

The process evaluation will use mixed methods (qualitative and quantitative, see Table 1), specifically:

1. Audio-recording of participant recruitment sessions (internal pilot study only), self-management teaching appointments and self-management support phone calls;
2. Qualitative interviews with randomised women from both trial groups, healthcare professionals who recruit to the trial and deliver pessary self-management and clinic-based pessary care, and women who decline to take part in the main trial but who are willing to be interviewed;
3. Checklist completion by health professionals on self-management teaching sessions and 2-week follow-up phone calls;
4. Process data (both quantitative and qualitative) embedded within main trial data collection. For example, an open question within the participant-completed questionnaires, and two measures of self-efficacy.

**Table 1:** Standard Protocol Items: Recommendations for Interventional Trials (SPIRIT) figure for the study

	Enrolment	Allocation	Follow-ups		Close out	On completion of all recruitment at a centre
Timepoint	-t1	0	6 months	12 months	18 months	variable
**Screening and eligibility**	X					
**Informed consent**		X				
**Interventions:** **Clinic-based care** **Self-management**						
**ASSESSMENTS**						
**Qualitative**						
Audio-recording of recruitment sessions	X					
Interviews with randomised women (both groups)		X			X	
Interviews with non-randomised women who use a pessary as treatment		X			X	
Interviews with health professional recruiters and those who deliver the intervention						X
Open question with main trial questionnaires		X	X	X	X	
**Quantitative**						
General Self-Efficacy		X			X	
Pessary Confidence Questionnaire		X	X	X	X	
Quality of life		X	X	X	X	
Pessary Use Questionnaire		X	X	X	X	
Checklist completion about self-management teaching appointment		X				
Recording sample of self-management teaching appointments		X				
Checklist completion for 2-week follow-up phone call		X (2 weeks after teaching)				
Recording sample of 2-week follow-up calls		X (2 weeks after teaching)				

### Internal pilot

The objective of the internal pilot study is to ensure the trial can recruit, randomise and retain sufficient numbers of participants whilst delivering the intervention as planned. Based on work by the QuinteT group [6, 12–16], we aim to audio-record 12–18 initial recruitment discussions with women, as part of the internal pilot study, to determine how the information about potential participation in the TOPSY study is delivered and discussed (two to three women in each of the six internal pilot centres). We expect these discussions to last between 10 and 15 min. Potential participants will receive a one-page participant information leaflet about the audio-recording of the initial recruitment discussion from a delegated member of the local clinical TOPSY research team. If willing to have their recruitment discussion recorded, written consent will be gained prior to the session commencing. If women do not want to take part, the initial recruitment discussion can still take place, but it will not be audio-recorded. If more than one person undertakes recruitment at any of the pilot centres, and they differ in professional background (e.g. research nurse and consultant), recruitment recordings will aim to reflect this diversity in professional background of recruiter. Sessions will be recorded using small, unobtrusive digital recorders. All six pilot centres will be asked to start recording recruitment sessions as soon as possible after the start of recruitment at their centre.

A subset of the interviews will be undertaken within the internal pilot study and also analysed within the main process evaluation data. Approximately five (of the 36 randomised interviews) and five of the interviews with women who decline randomisation but who are willing to be interviewed will be undertaken within the internal pilot study. The internal pilot interview analysis will focus on the subset of data within the interviews where women talk about trial recruitment. A recruiter from each of the internal pilot study centres will be asked to take part in an interview that focusses on recruitment processes. Approximately six of the self-management teaching appointments and follow-up phone calls will be recorded (one per pilot centre) and analysed with a focus on fidelity to the intervention delivery protocol during the pilot phase.

Ongoing analysis of all the information about recruitment and fidelity will support feedback to centres, and training of new centres, about the optimal way to recruit women to the study and deliver the intervention as planned.

### Recruitment and consenting processes

#### Audio-recordings

The Participant Information Leaflet for the TOPSY trial informs women that, if they are allocated to the self-management group, a self-management teaching appointment or a follow-up call may be recorded with their consent. Within the main trial consent form women are asked to indicate ‘yes/no’ to these recording by initialling the relevant box. Approximately 30 self-management teaching sessions and 30 self-management follow-up telephone calls will be recorded to achieve recordings across all participating centres.

#### Interviews with randomised women

The main trial Patient Information Leaflet outlines that some women will be invited for interview. The trial consent form again asks women to indicate (‘yes/no’) if they would be willing to be contacted about the interview study.

Women who are willing to be contacted about the interview study will be purposively sampled based on group allocation, age, pessary user status and regional spread. Pessary user status is differentiated between new users, those who used a pessary for 3 months or less, and existing users, women who have used a pessary for more than 3 months. Pessary user status is one of the minimisation criteria for the trial. The aim is to interview 36 women in total, 18 from each trial group. It is not possible to blind participants or the process evaluation researcher to the woman’s group allocation as both will know if the woman has received a self-management teaching appointment. The process evaluation researcher will post out the Interview Participant Information Leaflet and will call the woman a few days later to discuss their possible participation in the interview study. An additional consent form is signed for the interview study prior to the first interview.

#### Interview study with non-randomised women

Only women who are invited to take part in the trial in the clinic (as opposed to those who have information posted to them), and decline in the clinic, will be asked to take part in this component of the process evaluation. When women decline trial participation i.e. non-randomised women, they will be asked if they are willing to take a recruitment pack away for an interview study with non-randomised women. Those who indicate that they are willing will be given a recruitment pack in clinic. The recruitment pack will contain an introductory letter, a Participant Information Leaflet, an expression of interest form, a consent form and two reply-paid envelopes. Participants who choose to return the expression of interest form will be contacted by a member of the research team to answer any questions, go over the consent process and arrange a baseline telephone interview. Participants are asked to sign and return the consent form prior to the telephone interview. The aim is to interview 20 non-randomised women.

#### Healthcare professional interviews

During site initiation visits, healthcare professionals who are identified as being part of the Local TOPSY research team will be advised that they may be approached and invited to take part in an interview as part of the TOPSY study. All contact details for the Local TOPSY research team will be collected prior to the centre being opened to recruitment and as part of the delegation log. The TOPSY process evaluation researcher will contact healthcare professionals who recruit to the trial and/or deliver the intervention (approximately *n* = 34; one recruiter and one intervention deliverer for each trial site) to invite them to participate in the interview by sending them a Participant Information Leaflet. The researcher will answer any questions they might have and provide a consent form. Willing healthcare professionals will be asked to return the consent form to the TOPSY Team. Once written consent is obtained, a suitable date and time for telephone interview will be arranged.

### Data collection

The different components of the process evaluation data collection are described below.

#### Audio-recording of recruitment discussions

Where potential participants consent for their recruitment discussion to be recorded, the recruiter will be asked to record the discussion. This data will be used to highlight any barriers or facilitators to recruitment so that recruitment can be maximised.

#### Audio-recording of self-management teaching appointments and self-management support telephone calls and checklist completion

The aim is to audio-record 30 self-management teaching appointments and 30 2-week follow-up phone calls (at least one self-management teaching appointment and one phone call from each centre). Small digital recorders will be placed, with the agreement of the woman and healthcare professional, in the consulting room or attached to the phone to record all instruction and support given. Consent for these recordings is included within the main trial consent form and will be checked verbally before each recording. Variance across the sample will be aimed for in treating healthcare professional (nurse/physiotherapist/doctor) and women’s age. Checklists, that aim to assess fidelity, will be completed by the health care professional involved in the session/call for all self-management teaching appointments and all 2-week follow-up calls. The recordings and checklists will provide information about fidelity of intervention delivery.

#### Interviews with randomised women

Thirty-six women will be recruited (18 in the self-management group and 18 in the clinic-based care group). Purposive sampling will aim for variance in age, treating healthcare professional (nurse/physiotherapist/doctor), and centre type (outpatient/community/primary care). Interviews will be semi-structured and face-to-face and occur at randomisation and 18 months post-randomisation (where the primary outcome is measured). The interviews will explore perspectives on recruitment (baseline), symptoms and quality of life (baseline)/ change in symptoms and quality of life (18 months), experience and acceptability of self-management or clinic-based care (18 months), adherence to the allocated trial group (18 months), and contextual factors that are perceived to interact with the effectiveness of the intervention (18 months). Where participants have crossed over to receive treatment offered in the group to which she was not randomised, reasons for doing so will be explored during the 18-month follow-up interview.

All interview schedules will be developed with our Patients and Public Involvement (PPI) co-applicant and other PPI representatives. All interviews will be digitally recorded.

#### Interviews with women who decline randomisation

Twenty women who are potential participants for the trial and do not consent to randomisation, but who do consent to taking part in an interview, will be interviewed at baseline by telephone using a semi-structured interview schedule. If women consent to future participation in the interview study they will also be interviewed by phone at 18 months. Interviews will focus on reasons for declining trial participation (baseline); symptoms and quality of life (baseline)/ change in symptoms and quality of life (18 months); treatment received for prolapse (18 months); and contextual factors that may interact with future service implementation (baseline and 18 months).

#### Interviews with healthcare professionals who recruit to the trial and deliver the interventions

At least one healthcare professional who has delivered the self-management and/or clinic-based care intervention at each centre and a recruiter to the TOPSY trial will be given an information leaflet and invited to take part in a telephone interview study. Sampling will aim for diversity of professional group both for recruitment and for delivery. Recruiters and those who deliver interventions from the pilot centres will be interviewed at the end of the pilot phase. All other interviews will take place after trial recruitment is complete. Interviews will be semi-structured, last approximately 30 min and be undertaken by telephone. For recruiters, interviews will focus on factors that influence the identification of potential participants and recruitment, including service structures, contributing to maximising recruitment For those who have been involved in delivering clinic-based pessary care and/or self-management, interviews will focus on experiences of delivering self-management/clinic-based care, including variance in delivery and reasons for the variance, and contextual factors that were perceived to impact upon delivery. The objective here is to understand fidelity and contextual factors.

#### Process data embedded within main trial data collection

Within the main trial participant completed questionnaire there is a Pessary Use questionnaire within which there an open question that assesses women’s experience of their trial group (self-management or clinic-based care). Analysis of the responses to this question will support understanding of women’s experience of, and adherence to, their trial group.

The main trial questionnaire also contains the General Self-efficacy Questionnaire [17] and a pessary confidence questionnaire, developed specifically for the TOPSY study (described in detail in the trial protocol). These quantitative data will be analysed as part of the process evaluation analysis to explore the mediating influence of self-efficacy on quality of life.

### Data analysis

All analyses will be conducted according to a process evaluation analysis plan. The analysis plan will be approved by the Trial Steering Committee.

#### Qualitative data analysis

Initially, each individual data source will be analysed separately, and subsequently, findings will be synthesised across data sources. Qualitative data sources will be transcribed verbatim and entered into NVivo 12 (QSR International Pty Ltd., 2018. NVivo qualitative data analysis Software) for data management. Ten percent of transcripts within each data source will be coded independently by two analysts to assess for consistency in coding. All of the analysis described below will be undertaken by the process evaluation subgroup of grant-holders. Analysis will not be shared with the wider grant-holding group until the main trial findings are revealed. The process evaluation researcher will be blinded to the result of the clinical effectiveness findings during all stages of the analysis process described here.

The following qualitative data will be analysed using the Framework Approach to analysis [18]: audio-recordings of recruitment sessions; interviews with randomised women, women who declined randomisation and healthcare professionals; and the open question within the questionnaire.

Framework Analysis will move through stages of data management, descriptive analysis and finally onto interpretive and explanatory analysis.

For each individual dataset, the recommended Framework stages will be followed:

1. Data management

Familiarisation of the researcher with the data is the first step towards analysis. In keeping with Framework Analysis methods [18], the initial thematic framework for each dataset will be developed from the research questions, the data collection tools (e.g. interview schedules), themes that have arisen iteratively from the familiarisation process; discussion with co-applicants, and PPI consultation. In this way, the initial thematic framework contains both deductive and inductive elements. The thematic framework will then be inductively developed with the women’s voices shaping the analysis. Involving PPI representatives and the qualitative analysis team throughout will ensure that the thematic framework will be reviewed routinely and alternative ways of analysing the data considered.

2. Abstraction and interpretation

Abstraction and interpretation will occur first for each individual dataset and then the datasets will be combined. During this stage, categories and linkages within each dataset will be developed through comparison of tables and analytic memos. This process will be repeated for each of the purposes of the process evaluation to develop one overarching matrix for each purpose [19], e.g. bringing the tables together that focus on maximising recruitment into one matrix. Explanation will aim to bring the data together to interpret why the data have come together in the specific way that is presented.

Audio-recordings of the self-management teaching appointments and the self-management follow-up phone calls will be analysed by developing a structured analytic grid using the intervention protocols and the theory underlying the protocols. The grid will assess for key features within the teaching appointments and phone calls, for example, whether the healthcare professional teaching self-management offer the woman an opportunity to practice taking the pessary out and replacing it. The grid will contain explicit guidance as to what codes have to be applied in what circumstances. Coded data will then be subject to quantitative descriptive and interpretive analysis.

#### Quantitative data analysis for the process evaluation

Data collected from the pessary use questionnaire, general self-efficacy scale and the pessary confidence questionnaire will be analysed using quantitative methods. Data from the self-management appointments and 2-week follow-up calls (checklists and recordings) will also be analysed quantitatively with a particular focus on fidelity, such as the percentage of women who have been able to (1) remove and (2) replace their pessary themselves.

All process evaluation quantitative analyses will be conducted according to the pre-specified quantitative elements of the process evaluation analysis plan where possible using Stata version 15. All quantitative data will be described with the appropriate descriptive statistics: mean and standard deviation (SD) for continuous outcomes (or medians and interquartile range for skewed data), and counts and percentages for dichotomous and categorical outcomes.

We will undertake a statistical mediation analysis to investigate the extent to which any observed effect of self-management on women’s quality of life (measured using PFIQ-7 [20] at 18 months) is mediated by self-efficacy. Single-mediator models will individually assess the General Self-Efficacy scale and items from pessary confidence questionnaire as potential mediating factors.

#### Triangulation of process evaluation analysis

The quantitative and qualitative process evaluation analysis will be brought together using triangulation [19]. A matrix will be developed for each process evaluation concept (e.g. a matrix will focus on fidelity to the self-management intervention). The matrix will bring together the data sources and the relevant concept. The agreement/disagreement across sources will be documented.

A process evaluation management group oversees all aspects of the process evaluation, including the analysis. The group consists of the Process Evaluation Researcher, the Process Evaluation Lead, a clinical co-applicant and a PPI representative. The PPI representative will be involved in the analysis and interpretation of the data.

## Discussion

The TOPSY study will provide a novel comparison, from multiple methods, between self-management and clinic-based pessary care in the UK for women with pelvic organ prolapse using a vaginal pessary. Those perspectives will support an understanding of pessary self-management from an effectiveness, patient, healthcare professional and economic viewpoint. The process evaluation specifically will support new understanding of the contextual features that may influence intervention delivery and implementation in clinical practice and within women’s lives, as well as support and deepen our understanding about trial recruitment.

In this process evaluation, we have aimed to build on reports of contemporary process evaluation design [1] and good practice in trial recruitment [6, 12–16]. We plan to examine recruitment processes from multiple perspectives to better understand local structural elements of this multi-centre RCT with a view to maximising overall trial recruitment. It will be interesting to explore whether, or not, this embedded recruitment work enhances trial recruitment. The inclusion of eligible women who have declined trial participation is another distinctive part of this process evaluation. This inclusion is anticipated to strengthen our understanding of how women feel about participating in an RCT regarding vaginal pessary management and the acceptability of recruitment processes and materials. It further offers the opportunity for a qualitative comparison of participant characteristics of randomised trial participants and women declining participation.

Having built the process evaluation into the study design from the outset will strengthen the overall outcomes. In its comprehensiveness, the process evaluation looks at what works for whom and how by including patient and healthcare perspectives. Local structural differences are recorded in various ways providing valuable contextual information which allows a deeper analysis of the mechanisms of the implementation. Some researchers have questioned the suitability of process evaluations as part of an RCT as they see them as two ontologically opposing elements [21, 22]. Whilst it is challenging to separate specific mechanisms from each other to determine exactly which ones have an impact on intervention outcomes, not acknowledging the importance of contextual factors on the potential success of an intervention, dismisses the opportunity to find potential solutions and avenues for adjustments to potential obstacles to successfully implement a healthcare intervention.

### Current status of process evaluation

Recruitment and data collection are ongoing across all centres across the UK. Recruitment started (to the trial and process evaluation) in May 2018 and is expected to continue until January 2020. Data collection and recruitment for the recruitment discussion recordings, which was limited to internal pilot centres, is complete. The pilot study was completed on 16th November 2018. It is anticipated that data collection for the process evaluation will be complete by July 2021, 18 months after the last randomisation.

## Trial status

The first participant to the trial was randomised on the 16th May 2018. The first participant for the randomised interview study was consented on 14 June 2018. The first participant for the non-randomised interview study was consented on 29 August 2018. Recruitment and consenting for audio-recordings of the self-management teaching appointments and follow-up calls is ongoing and is anticipated to be completed February 2020. Recruitment and consenting for interviews with healthcare professionals recruiting to TOPSY and delivering the TOPSY intervention is ongoing and anticipated to be completed in April 2020. Recruitment to the trial will continue until January 2020. Data collection for the trial will continue until 2021.The protocol version 5 presented here received ethical approval on 25th July 2019.

## Data Availability

Data from the study will be made publicly available at the end of the trial, on request. Requests will be subject to approval by the Chief Investigator, the PMG, the TSC and relevant ethical bodies. All data requests should be submitted to the corresponding author for consideration. Access to anonymised data may be granted following review.

## Abbreviations

MRC: Medical Research Council
PPI: Patient and Public Involvement
RCT: Randomised controlled trial
SD: Standard deviation
